# Reduced expression of stearoyl-CoA desaturase-1, but not free fatty acid receptor 2 or 4 in subcutaneous adipose tissue of patients with newly diagnosed type 2 diabetes mellitus

**DOI:** 10.1038/s41387-018-0054-9

**Published:** 2018-09-07

**Authors:** Kálmán Bódis, Sabine Kahl, Marie-Christine Simon, Zhou Zhou, Henrike Sell, Birgit Knebel, Andrea Tura, Klaus Strassburger, Volker Burkart, Karsten Müssig, Daniel Markgraf, Hadi Al-Hasani, Julia Szendroedi, Michael Roden, for the GDS Study Group, A. E. Buyken, B. Belgardt, G. Geerling, H. Al-Hasani, C. Herder, A. Icks, J. Kotzka, O. Kuss, E. Lammert, D. Markgraf, K. Müssig, W. Rathmann, J. Szendroedi, D. Ziegler, M. Roden

**Affiliations:** 1 Institute for Clinical Diabetology, German Diabetes Center, Leibniz Center for Diabetes Research at Heinrich Heine University, Düsseldorf, Germany; 2 German Center for Diabetes Research (DZD), München-Neuherberg, Germany; 3 Division of Endocrinology and Diabetology, Medical Faculty, Heinrich Heine University, Düsseldorf, Germany; 4 Institute for Clinical Biochemistry and Pathobiochemistry, German Diabetes Center, Leibniz Center for Diabetes Research at the Heinrich Heine University, Düsseldorf, Germany; 5 Metabolic Unit, Institute of Biomedical Engineering, National Research Council, Padua, Italy; 6 Institute for Biometrics and Epidemiology, German Diabetes Center, Leibniz Center for Diabetes Research at Heinrich Heine University, Düsseldorf, Germany

## Abstract

**Background:**

In subcutaneous adipose tissue (SAT), higher *stearoyl-CoA desaturase-1* (*SCD1*) expression has been related to improved insulin sensitivity in thiazolidinedione-treated type 2 diabetes mellitus patients. In animal models, deficiency of the *free fatty acid receptor* (*FFAR*) *2* associated with higher and *FFAR4*-deficiency with lower insulin sensitivity. We hypothesized that increased FFAR2 expression and reductions in FFAR4 and SCD1 expression in SAT of type 2 diabetes mellitus patients associate positively with insulin resistance and impaired beta cell function.

**Methods:**

Twenty-five type 2 diabetes mellitus patients and 25 glucose-tolerant humans (CON) matched for sex, age, and BMI underwent mixed-meal tests to assess insulin sensitivity (OGIS) and beta cell function (ΔAUC(C-peptide)_0–180 min_/ΔAUC(glucose)_0–180 min_) in a cross-sectional study. Gene and protein expression of SCD1 and FFAR2/4 were quantified in SAT biopsies.

**Results:**

Insulin sensitivity was 14% and beta cell function 71% (both *p* < 0.001) lower in type 2 diabetes mellitus patients. In type 2 diabetes mellitus, *SCD1* mRNA was fivefold (*p* < 0.001) and protein expression twofold (*p* < 0.01) lower. While FFAR2/4 mRNA and protein expression did not differ between groups, FFAR2 protein levels correlated negatively with beta cell function only in CON (*r* = −0.74, *p* < 0.01). However, neither SCD1 nor FFAR2/4 protein expression correlated with insulin sensitivity in both groups.

**Conclusions:**

Type 2 diabetes patients have lower SCD1, which does not associate with insulin resistance. Only in non-diabetic humans, FFAR2 associated with impaired beta cell function.

## Introduction

Type 2 diabetes is characterized by insufficient insulin secretion to compensate for peripheral insulin resistance^1^. Studies in animal models and humans implicate free fatty acid receptors (FFAR), also known as G-protein coupled receptors (GPR), as receptors for non-esterified fatty acids (NEFA) in the pathogenesis of beta cell dysfunction and progression to insulin resistance and type 2 diabetes mellitus^2,3^.

FFAR2 and FFAR4 (also known as GPR43 and GPR120) serve as receptors for acetate and long-chain fatty acids (FA)^4,5^, respectively, and are supposed to contribute to the regulation of glucose homeostasis through FA signaling pathways^6^. Recent studies provided evidence that *FFAR2*-deficient mice on high-fat diet are protected from the increase in body fat mass and dyslipidemia, accompanied by increased insulin sensitivity (IS)^7^. In addition, in mouse islets *FFAR2* gene expression was increased during the insulin-resistant phase of pregnancy^8,9^. One translational study further identified gene expression of *FFAR2* in mouse and human islets, and suggested FFAR2 to mediate inhibition of insulin secretion by coupling to G_i_-type G proteins^3^. Beta cell-specific deletion of *FFAR2* in another mouse model led to increased insulin secretion and improved glucose tolerance^3^. These findings in beta cells from mouse models and human in vitro studies point to an involvement of FFAR2 in maintaining glucose homeostasis. Adipose tissue (AT) expansion in obesity associates with insulin resistance and progressive immune cell infiltration in AT^10^. Pro-inflammatory cytokines activate lipolysis^11^ causing dyslipidemia^12^, lipid-induced insulin resistance in peripheral tissues^13^, and impairment of beta cell function^14^. In contrast, FFAR2 knock out mice were protected from high-fat diet-induced AT inflammation and obesity^7^. Thus, FFAR2 may serve as a potential target for diabetes prevention strategies via inhibition of lipid-induced insulin resistance.

A previous study showed that FFAR4 activation by omega-3 fatty acid protected human islets from palmitate-induced apoptosis, whereas FFAR4 knock out attenuated omega-3 fatty acid-related anti-apoptotic effects^15^. Compared to wild-type mice, high-fat fed *FFAR4*-deficient mice developed more severe obesity, liver fat accumulation, and insulin resistance^16,17^. However, these findings were accompanied by lower *stearoyl-CoA desaturase-1* (*SCD1*) gene expression in AT^18^. In murine models, four isoforms (SCD1, SCD2, SCD3, and SCD4) have been identified, whereas humans express only two Δ9 desaturases (SCD1 and SCD5). Our study focused on SCD1 as the most highly expressed SCD isoform in AT. SCD1 in AT facilitates the protective conversion of lipotoxic lipid species (saturated into monounsaturated FA). Circulating palmitoleate, an AT derived product of SCD1, increased insulin signaling in both skeletal muscle and the liver, increased insulin secretion from beta cells, and improved whole-body glucose uptake in mice^19^. Furthermore, palmitoleate treatment reduced cytokine expression in cultured adipocytes^19^. SCD1 in AT facilitates the last step of de novo lipogenesis and induces incorporation of FA into triglycerides (TG), both associating positively with whole-body insulin sensitivity. Accordingly, thiazolidinedione treatment promoted TG esterification in cultured adipocytes^20^ and increased *SCD1* gene expression in subcutaneous adipose tissue (SAT) with subsequent improvement of IS in patients with type 2 diabetes mellitus, suggesting a potential role of SCD1 in AT on systemic glucose homeostasis^21^.

Although FFAR2/4 and SCD1 seem to be involved in maintaining glucose homeostasis in mice^3,16,17,22^, the relevance of their expression in human SAT for glucose homeostasis has not yet been elucidated. FFARs and SCD1 are expressed in various tissues, but might be especially important in AT due to its prominent role in lipid turnover. Here, we hypothesized that increased FFAR2 expression and reduced FFAR4 and SCD1 expression in SAT of patients with type 2 diabetes mellitus in the fasted state associate positively with insulin resistance and inversely with beta cell function. Furthermore, we hypothesized that increased FFAR2 and reduced FFAR4 expression in AT of type 2 diabetes patients associate with parameters of dyslipidemia. Finally, we hypothesized that higher SCD1 expression in AT of type 2 diabetes patients associates negatively with high-sensitivity C-reactive protein (hsCRP) in plasma. To this end, we analyzed FFAR2 as well as FFAR4 and SCD1 mRNA and protein expression in SAT of 25 metabolically well-characterized patients with newly diagnosed type 2 diabetes mellitus and 25 age-matched, sex-matched, and BMI-matched glucose-tolerant humans (CON).

## Materials and methods

### Study participants

The study population comprised 25 patients with recently diagnosed type 2 diabetes mellitus and 25 age-matched, sex-matched, and BMI-matched CON. All participants gave their written informed consent before inclusion into the study (ClinicalTrial.gov registration no: NCT01055093), which was performed according to the Declaration of Helsinki and approved by the ethics board of Heinrich Heine University, Düsseldorf, Germany. Participants were recruited via general practitioners, internet, or advertisements in newspapers. For three days prior to each visit, participants refrained from physical activity and alcohol ingestion and fasted for 10 h on the day before the metabolic studies. Exclusion criteria comprised medical history of acute or chronic diseases including cancer, insulin or thiazolidinedione treatment, medication affecting the immune system and/or a HbA_1c_ > 9.0% (75 mmol mol^−1^), diabetes other than type 2 diabetes mellitus. Patients with type 2 diabetes mellitus were treated with metformin only (*n* = 15), sulfonylurea only (*n* = 2), metformin and sulfonylurea (*n* = 2), glucagon-like peptide-1 receptor agonist and metformin (*n* = 1), or diet only (*n* = 5). They withdrew their oral glucose-lowering medication for at least 3 days before all measurement to exclude acute effects on glucose metabolism^23^. All patients with type 2 diabetes mellitus also participated in the baseline cohort of the ongoing German Diabetes Study (GDS), a prospective observational study investigating the natural course of recently diagnosed diabetes and the development of diabetes-associated complications. The study design and cohort profile of the GDS are described in detail elsewhere^23^. Age-matched, sex-matched, and BMI-matched glucose-tolerant participants were recruited as control group (CON).

### Mixed-meal test (MMT)

All participants underwent a standardized MMT to assess whole-body IS and beta cell function. For the MMT, each participant consumed 360 ml of Boost High Protein (Nestlé Nutrition, Vervey, Switzerland) containing 41 g of glucose, 9 g of fat, and 23 g protein before 10 am within 5 min followed by defined blood sampling for specific parameters described elsewhere^23,24^. Dynamic IS was assessed by the oral glucose insulin sensitivity index (OGIS), which allows calculating whole-body IS during both oral glucose tolerance test (OGTT) and MMT, provided that the dose of glucose administered during the test is taken into account^25,26^. Beta cell function was assessed from incremental AUC of plasma glucose, insulin, and C-peptide concentrations during MMT. The insulinogenic index from ΔAUC(C-peptide)_0–180 min_/ΔAUC(glucose)_0–180 min_, and ΔAUC(insulin)_0–180 min_/ΔAUC(glucose)_0–180 min_ was used to describe insulin secretion in relationship to glucose as a measure of beta cell function^27^.

### Oral glucose tolerance test (OGTT)

All CON underwent a 75 g-OGTT (Accu-Chek Dextro O.G-T., Roche, Basel, Switzerland) after at least 10 h overnight fasting to assess glucose tolerance and exclude participants with (pre-) diabetes mellitus. Blood samples were taken at time points −5, 30, 60, 120, and 180 min and glucose tolerance was categorized according to internationally accepted criteria^28^.

### Laboratory analyses

Plasma glucose, HbA_1c_, NEFA, high-density lipoprotein (HDL) cholesterol, low-density lipoprotein (LDL) cholesterol, TG and hsCRP, insulin, and C-peptide were measured as previously described^23^. Plasma glucagon was measured using radioimmunoassay (Millipore, St. Charles, MO, USA).

### Adipose tissue analyses

A biopsy was obtained from abdominal SAT at the level of umbilicus by needle suction technique after administration of local anesthesia (5–10 ml of 1% lidocaine) under fasting conditions. Fat tissue specimen were immediately frozen in liquid nitrogen, and stored −80 °C until analysis. For analyses of mRNA expression, total RNA was isolated (miRNeasy Mini Kit, Qiagen, Hilden, Germany) including on-column DNase digestion. For gene expression analyses, RNA quantity and quality were determined by Nanodrop (Peqlab, Erlangen, Germany) and RNA 6000 Nano Kit (Agilent Technologies, Böblingen, Germany). The complementary DNA equivalent of 20 ng RNA was analyzed by RT-qPCR using predesigned assays with gene-specific hydrolysis probes (*FFAR2*: Hs00271142_s1; *FFAR4*: Hs00699184_m1; *SCD1*: Hs01682761_m1; *peptidylprolyl isomerase A (PPIA)*: Hs04194521_s1; Thermo Fisher Scientific, Darmstadt, Germany). Data were analyzed for relative expression differences using *PPIA* as reference gene with standard *C*_t_ method as previously described^29^. For protein expression analyses, reagents for SDS-PAGE were supplied by GE Healthcare (Freiburg, Germany). Complete protease inhibitor cocktail and PhosStop phosphatase inhibitor cocktail were provided by Roche (Mannheim, Germany). All other chemicals were of the highest analytic grade commercially available and purchased from Sigma-Aldrich. The following antibodies were used: anti-G-protein coupled receptor (GPR) 43 (FFAR2) (sc-293202) from Santa Cruz (Dallas, TX, USA), anti-GPR120 (FFAR4) (NBP1-00858) from Novus Biologicals (Abingdon, UK), anti-SCD1 (ab39969), and anti-actin (ab6276) from Abcam (Cambridge, UK). Horseradish peroxidase (HRP)-conjugated goat anti-rabbit and goat anti-mouse IgG antibodies were supplied by Promega (Mannheim, Germany). AT biopsies were lysed in a buffer containing 50 mmol l^−1^ HEPES, pH 7.4, 1% Triton X-100, complete protease inhibitor, and PhosStop phosphatase inhibitor cocktail. After incubation for 2 h at 4 °C, the suspension was centrifuged at 10,000×*g* for 15 min. Thereafter, 10 µg of the lysates were separated by SDS-PAGE using gradient horizontal gels and transferred to polyvinylidene fluoride filters in a semidry blotting apparatus. Filters were blocked with Tris-buffered saline containing 0.1% Tween and 5% nonfat dry milk and subsequently incubated overnight with a 1:1000 dilution (1:40,000 for anti-actin) of the appropriate antibodies. After washing, filters were incubated with secondary HRP-coupled antibody and processed for enhanced chemiluminescence detection using Immobilon HRP substrate (Millipore, Billerica, MA, USA). Signals were visualized and evaluated on a ChemiDoc workstation (Bio-Rad Laboratories, Munich, Germany).

### Statistical analyses

Results are given as median [first and third quartiles] or mean ± SEM. Data were compared using Mann–Whitney *U* test for unpaired samples to determine differences between groups. Relations between variables were investigated using Spearman rank correlation analyses. The total AUC for a specific variable was calculated as the integral of the time course of such variable during the test, while the incremental AUC (ΔAUC) was calculated by subtracting the basal area from the respective total AUC. All statistical tests were two-sided and a *p*-value ≤ 5% was accepted to indicate significant differences. All statistical analyses were performed using SPSS for Windows 23.0 (SPSS Inc., Chicago, IL, USA). All graphs were generated using GraphPad Prism, Version 7.01 (GraphPad Software, Inc., La Jolla, CA, USA).

## Results

### Participants’ characteristics

CON and patients with type 2 diabetes mellitus had similar age, BMI, waist circumference, waist-to-hip ratio, and relative body fat mass (Table 1). Patients with type 2 diabetes mellitus had 34% higher fasting plasma glucose, 28% higher fasting insulin, and 29% higher fasting C-peptide. Additionally, serum hsCRP levels were 56% higher in patients with type 2 diabetes mellitus compared to CON. Furthermore, HDL cholesterol was 25% lower and systolic blood pressure 8% higher in patients with type 2 diabetes mellitus (Table 1). Of note, one female patient with type 2 diabetes was in the luteal phase. However, exclusion of this patient did not affect the results. All other females investigated were either postmenopausal or studied in the follicular phase (day 1–14) of their menstrual cycle. Furthermore, one woman in the follicular phase was controlled by an oral contraceptive.

**Table 1:** Characteristics of study population

Variable	CON	T2D	*p*
Female/male [*n*]	6/19	6/19	
Age [years]	51 [42; 60]	49 [43; 58]	0.84
Weight [kg]	98 [91; 98]	99 [88; 112]	0.61
BMI [kg* m^−2^]	33 [27; 41]	34 [27; 37]	0.81
HbA_1c_ [%]	5.3 [5.1; 5.5]	6.1 [5.5; 7.0]	**<0.001**
HbA_1c_ [mmol* mol^−1^]	34 [32; 37]	43 [36; 52]	**<0.001**
Waist circumference [cm]	106 [99; 116]	107 [102; 118]	0.54
Hip circumference [cm]	111 [97; 127]	110 [102; 118]	0.97
WHR	0.98 [0.90; 1.04]	0.99 [0.93; 1.04]	0.54
Body fat [%]	34 [27; 42]	33 [28; 39]	0.87
Lean body weight [kg]	66 [57; 74]	65 [58; 72]	0.87
Systolic blood pressure [mmHg]	124 [114; 143]	144 [126; 159]	**<0.05**
Diastolic blood pressure [mmHg]	80 [69; 86]	80 [73; 94]	0.40
Fasting blood glucose [mmol* l^−1^]	4.6 [4.4; 4.7]	6.7 [5.7; 8.0]	**<0.001**
Fasting insulin [µU* ml^−1^]	11 [6; 17]	17 [11; 27]	**<0.05**
Fasting C-peptide [ng* ml^−1^]	2.3 [1.5; 3.1]	3.4 [2.0; 4.7]	**<0.05**
Fasting glucagon [pg* ml^−1^]	118 [98; 137]	110 [76; 138]	0.34
NEFA [µmol* l^−1^]	603 [431; 655]	481 [333; 652]	0.21
TG [mg* dl^−1^]	119 [82; 144]	117 [102; 252]	0.17
HDL cholesterol [mg* dl^−1^]	53 [43; 66]	38 [33; 46]	**<0.001**
LDL cholesterol [mg* dl^−1^]	135 [114; 156]	127 [111; 139]	0.21
hsCRP [mg* dl^−1^]	0.13 [0.07; 0.33]	0.34 [0.14; 0.72]	**<0.05**
OGIS_0–180 min_ [ml* min^−1^* m^−^^2^]	362 [349; 385]	311 [293; 343]	**<0.001**

Data are median [first; third quartile], two-tailed Mann–Whitney *U* test (*n* = 22–25 for CON; *n* = 16–25 for type 2 diabetes patients). Significant differences between groups (*p*-value ≤ 5%) are indicated by bold values.  Glucose-tolerant humans (CON), patients with type 2 diabetes mellitus (T2D), waist/hip ratio (WHR), diastolic and systolic blood pressure (BP), oral glucose insulin sensitivity index (OGIS). Triglycerides (TG), high-density lipoprotein (HDL) cholesterol, low-density lipoprotein (LDL) cholesterol, non-esterified fatty acids (NEFA), and high-sensitivity C-reactive protein (hsCRP) were analyzed in fasted state

### Mixed-meal test (MMT)

In patients with type 2 diabetes mellitus, the glucose concentrations were higher at all time points during the MMT and AUC(glucose)_0–180 min_ was also 31% higher (Table 2, Supplementary Figure 1a). Insulin at 30 min and C-peptide at 90, 120, and 180 min were higher in patients with type 2 diabetes mellitus vs CON, while AUC(insulin)_0–180 min_ and AUC(C-peptide)_0–180 min_ did not differ between groups (Table 2, Supplementary Figure 1b, c). AUC(NEFA)_0–120 min_, AUC(TG)_0–180 min_, and AUC(glucagon)_0–180 min_ also did not differ between groups (data not shown). In patients with type 2 diabetes mellitus, OGIS was 14% lower and beta cell function assessed from ΔAUC(C-peptide)_0–180 min_/ΔAUC(glucose)_0–180 min_ as well as from ΔAUC(insulin)_0–180 min_/ΔAUC(glucose)_0–180 min_ was 71% and 78% lower than CON, respectively.

**Table 2:** Characteristics of beta cell function in study population

Variable	CON	T2D	*p*
AUC 0–180 min			
AUC(glucose)_0–180 min_ [mmol* l^−1^ min^−^^1^]	842 [772; 901]	1 188 [1 080; 1 407]	**<0.001**
AUC(insulin)_0–180 min_ [nmol* l^−^^1^ min^−1^]	48 [25; 68]	49 [31;65]	0.77
AUC(C-peptide)_0–180 min_ [nmol* l^−1^ min^−1^]	350 [238; 405]	373 [290; 506]	0.17
Beta cell function			
ΔAUC(insulin)_0–180 min_/ΔAUC(glucose)_0–180 min_ [pmol* mmol^−1^]	680 [408; 1367]	174 [92; 228]	**<0.001**
ΔAUC(C-peptide)_0–180 min_/ΔAUC(glucose)_0–180 min_ [nmol* mmol^−1^]	46 [20; 78]	13 [8; 21]	**<0.001**

Data are median [first; third quartile], two-tailed Mann–Whitney *U* test (*n* = 13–25 for CON; *n* = 18–25 for patients with type 2 diabetes mellitus). Significant differences between groups (p-value ≤ 5%) are indicated by bold values. Glucose-tolerant humans (CON), patients with type 2 diabetes mellitus (T2D). Marker for beta cell function are ΔAUC(insulin)_0–180 min_/ΔAUC(glucose)_0–180 min_ in pmol* mmol^−1^ and ΔAUC(C-peptide)_0–180 min_/ΔAUC(glucose)_0–180 min_ in nmol* mmol^−1^

### Gene and protein expression levels

**Fig. 1:**
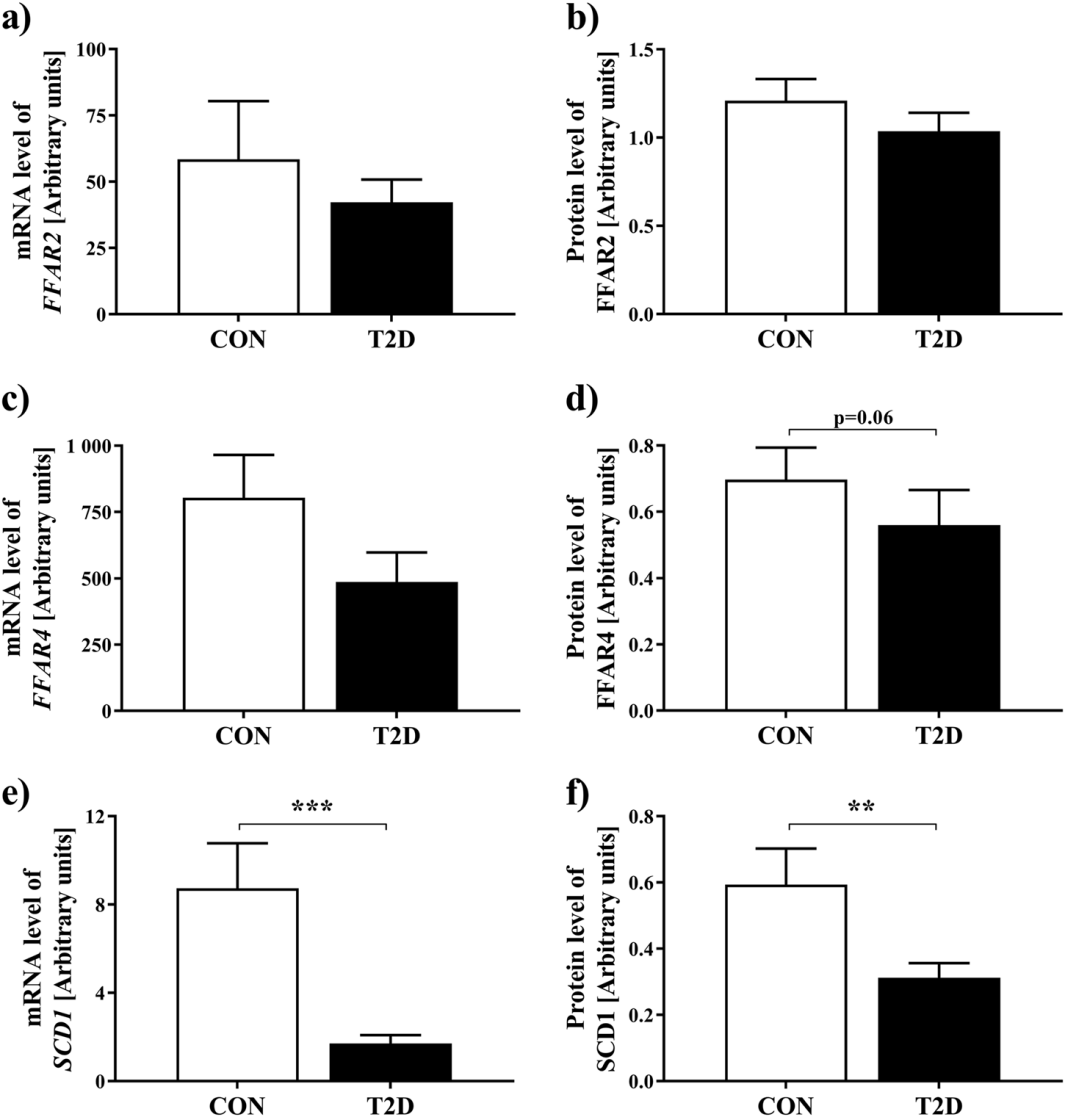
mRNA and protein expression analyses for genes involved in fatty acid metabolism Data are mean ± SEM (*n* = 23–25 for CON; *n* = 22–25 for patients with type 2 diabetes mellitus), two-tailed Mann–Whitney *U* test. **p < 0.01, ***p < 0.001. Data were analyzed for relative expression differences using *peptidylprolyl isomerase A (PPIA)* as reference gene with standard *C*_t_ method. Glucose-tolerant humans (CON), patients with type 2 diabetes mellitus (T2D). **a** mRNA expression levels of *free fatty acid receptor 2* (*FFAR2*), **b** protein expression levels of FFAR2, **c** mRNA expression levels of *free fatty acid receptor 4* (*FFAR4*), **d** protein expression levels of FFAR4, **e** mRNA expression levels of *stearoyl-CoA desaturase-*1 (*SCD1*), **f** protein expression levels of SCD1

*FFAR2* mRNA and protein expression were similar in type 2 diabetes mellitus patients compared to CON (Fig. 1a, b, Supplementary Figure 2a). *FFAR4* mRNA expression did not differ, while FFAR4 protein expression tended to be lower in patients with type 2 diabetes mellitus (Fig. 1c, d, Supplementary Figure 2b). Patients with type 2 diabetes mellitus had fivefold lower mRNA expression and twofold lower protein expression of SCD1 compared to CON (Fig. 1e, f, Supplementary Figure 2c).

### Correlation analyses

**Fig. 2:**
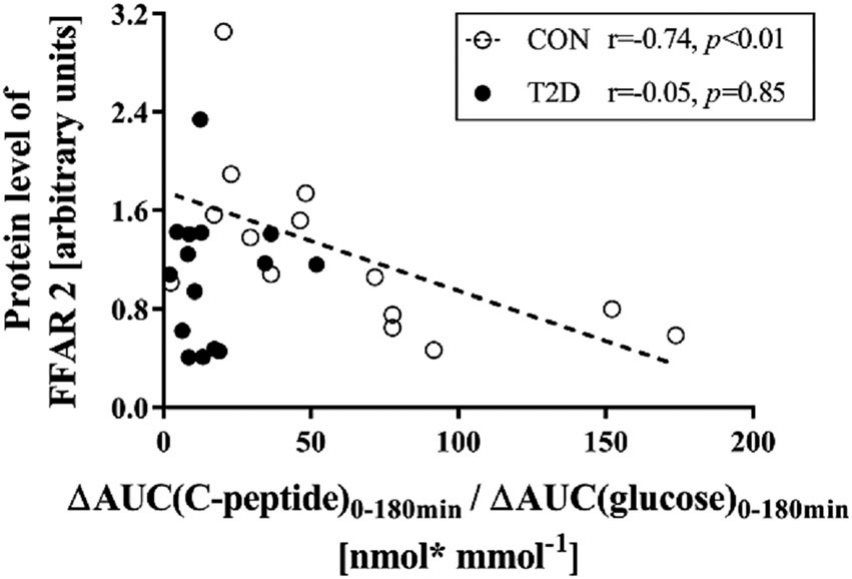
Association of protein expression levels of FFAR2 with beta cell function *r*-Spearman’s rank correlation coefficient. Glucose-tolerant humans (CON), patients with type 2 diabetes mellitus (T2D), free fatty acid receptor 2 (FFAR2), marker for beta cell function from ΔAUC(C-peptide)_0–180 min_/ΔAUC(glucose)_0–180 min_ in nmol* mmol^−1^

FFAR2 protein expression neither correlated with OGIS in type 2 diabetes mellitus patients nor in CON. Only in CON, protein expression levels of FFAR2 associated negatively with beta cell function, as assessed from ΔAUC(insulin)_0–180 min_/ΔAUC(glucose)_0–180 min_ (*r* = −0.78, *p* < 0.01) and ΔAUC(C-peptide)_0–180 min_/ΔAUC(glucose)_0–180 min_ (Fig. 2). Of note, fasted NEFA levels and AUC(NEFA)_0–120 min_ did not correlate with beta cell function. Only in patients with type 2 diabetes mellitus, protein levels of FFAR2 correlated positively with AUC(TG)_0–180 min_ (*r* = 0.48, *p* < 0.05) and negatively with HDL cholesterol (*r* = −0.43, *p* < 0.05). FFAR4 protein expression did not correlate with OGIS or beta cell function in both groups. Protein expression levels of FFAR4 associated negatively with fasting TG and AUC(TG)_0–180 min_ in patients with type 2 diabetes mellitus (*r* = −0.62 and *r* = −0.59, respectively, both *p* < 0.01), but not in CON (*r* = 0.15, *p* = 0.48 and *r* = 0.25, *p* = 0.26, respectively). FFAR2/4 did not associate with body weight, BMI, or relative body fat content in both groups.

SCD1 protein expression did not correlate with OGIS or beta cell function in both groups. However, SCD1 mRNA and protein levels associated positively with insulin sensitivity across the whole cohort (*r* = 0.38, *p* < 0.01 and *r* = 0.31, *p* < 0.05, respectively). SCD1 protein expression correlated positively with mRNA levels (*r* = 0.60, *p* < 0.001). In CON, protein levels of SCD1 associated negatively with fasting TG (*r* = −0.57, *p* < 0.01) and AUC(TG)_0–180 min_ (*r* = −0.52, *p* < 0.05). In patients with type 2 diabetes mellitus, these associations were absent (TG: *r* = −0.17, *p* = 0.44; AUC(TG)_0–180 min_: *r* = 0.05, *p* = 0.85). Only in patients with type 2 diabetes mellitus, SCD1 protein levels correlated negatively with hsCRP (*r* = −0.45, *p* < 0.05).

## Discussion

This study found no differences in FFAR2/4 mRNA and protein expression between patients with type 2 diabetes mellitus vs CON of similar body weight. Our findings are in contrast to results in mice, which might be biased by differences in body weight explaining a large part of their phenotypes^30^. We found markedly lower gene and protein expression of SCD1 in subcutaneous AT of patients with type 2 diabetes mellitus when compared with sex-matched, age-matched, and BMI-matched glucose-tolerant humans. Furthermore, FFAR2 protein expression correlated negatively with beta cell function in glucose-tolerant humans. However, this association was not found in patients with type 2 diabetes mellitus.

In mice, a recent study of whole-body or beta cell selective deletion of *FFAR2* also provided evidence that insulin secretion is increased, accompanied by improved glucose tolerance^3^. In the present study, FFAR2 expression did not differ between patients with type 2 diabetes mellitus and CON, while its protein levels negatively associated with beta cell function during MMT in CON. This points to a potential impact of the receptor on glucose homeostasis after food intake rather than in the fasted state. In addition, the association between *FFAR2-*deficiency and IS in mice^7^ was not confirmed in humans with or without diabetes in the present study, indicating that reported findings in FFAR2-deficient mice cannot directly be translated to humans. Although previous studies indicated an assumed role in AT lipolysis^11^ causing impaired beta cell function^14^, we did not find a significant correlation of NEFA levels either during fasting or after the MMT with beta cell function. However, suppression of NEFA concentrations after the MMT might not be an optimal method to assess AT lipolysis, because of the exogenous oral intake of lipids and the individual variability of lipid absorption. Thus, the absence of a significant correlation between suppression of NEFA levels after the MMT and beta cell function does not exclude effects of insulin-mediated adipose tissue lipolysis.

In humans, a deleterious non-synonymous mutation (p.R270H) that inhibits *FFAR4* signaling was found to associate with increased risk of obesity in a European population^16^. Of note, development of severe obesity, liver fat accumulation, and insulin resistance in *FFAR4*-deficient mice under high-fat diet^16,17^ were accompanied by lower *SCD1* gene expression^18^. We found no association between FFAR4 and body weight or differences in human FFAR4 expression between patients with type 2 diabetes mellitus and CON, but FFAR4 protein expression tended to be lower in type 2 diabetes mellitus. One possible explanation for the lack of differences between groups in FFAR4 expression might be that these receptors are dependent on acute increase in NEFAs triggering FFAR4 expression in AT. In the present study, fasted NEFA and TG levels did not differ between groups, mainly because our study only included patients with recently diagnosed and well controlled type 2 diabetes mellitus.

Interestingly, our study revealed markedly lower gene and protein expression of SCD1 in SAT of patients with type 2 diabetes mellitus. These findings are in line with the previously reported decrease in *SCD1* gene expression in FFAR4-deficient mice^18^ and support a role of SCD1 (Δ9 desaturase) in glucose homeostasis. Desaturases are key enzymes in converting saturated to unsaturated FAs by introducing a double-bond in the growing FA chain. Of note, the large prospective population-based Kuopio Ischaemic Heart Disease (KIHD) Risk Factor Study indicated that higher serum Δ5 desaturase activity associate with a lower risk for type 2 diabetes mellitus and increased Δ6 desaturase activity with a higher risk among middle-aged and older Finish men^31^. A putative mechanism behind the negative association of higher Δ5 desaturase activity with lower risk of type 2 diabetes mellitus may be the simultaneous improvement in IS^32^. In the European Prospective Investigation into Cancer (EPIC) and Nutrition-Potsdam Study, SCD1 activity (assessed from product-to-precursor ratios) of erythrocyte membrane was positively associated with risk of type 2 diabetes mellitus^33^. In agreement with our findings and previously reported beneficial effects of SCD1 in type 2 diabetes mellitus^21,34^, thiazolidinedione treatment led to increased *SCD1* expression in AT of patients with type 2 diabetes mellitus together with improved TG esterification and IS^20^. The mechanisms underlying insulin resistance are still not fully understood, but increased NEFA release from AT is generally recognized as an important factor for the development of insulin resistance^35,36^. Thus, dysfunctional AT as indicated by decreased SCD1 expression in type 2 diabetes mellitus compared to CON may contribute to the development of type 2 diabetes mellitus. Previously, we showed that an intravenous lipid infusion as well as a single oral fat load rich in long-chain polyunsaturated FAs can induce insulin resistance^29,37^. Especially, saturated FAs are thought to induce inflammation and insulin resistance^38–40^. Accordingly, *SCD1*-deficient mice exhibited increased inflammation^41^. In agreement with our results, AT-specific *SCD1* deletion in a mouse model induced glucose transporter 1 upregulation in AT, which was associated with increased tumor necrosis factor-alpha production^42^. The possible protective effect of SCD1 in CON is underlined by our observation that protein levels of SCD1 correlated negatively with hsCRP in patients with type 2 diabetes mellitus. Moreover, SCD1 protein levels in CON associated negatively with fasting TG and postprandial AUC(TG)_0–180 min_, respectively. The decrease in plasma TG levels with increasing SCD1 protein expression is in accordance with previous studies, where patients with type 2 diabetes mellitus exhibited enhanced TG esterification in SAT under thiazolidinedione treatment with subsequently increased *SCD1* gene expression^20^.

The strength of our study lies in the deep phenotyping of patients with type 2 diabetes mellitus and well-matched glucose-tolerant controls. However, the conclusions from our study are limited by the small sample size and the lack of muscle samples. Due to the nature of a cross-sectional design, this study also does not allow to draw conclusions as to causality or temporal relationships.

In conclusion, patients with recent onset type 2 diabetes mellitus have lower SCD1, but not FFAR2 or 4 expression in SAT compared to CON. Our findings suggest that SCD1 expression may be important in early development of type 2 diabetes mellitus, but is not as effective in modulating beta cell function as FFAR2. Our study implies that FFAR2 could negatively influence glucose homeostasis by decreasing beta cell function in CON. Thus, both FFAR2 and SCD1 may be potential treatment targets in diabetes prevention strategies.

### Data availability

To ensure data privacy of the study participants, the generated datasets of the currently still running GDS are not publicly available. Especially, since they are subject to national data protection laws and restrictions by the ethics committee. However, access to the data can be requested through an individual project agreement within the GDS.

### Disclaimer

The funding sources had neither influence on design and conduct of this study, collection, analysis, and interpretation of the data; nor on the preparation, review, or approval of this article.

## Electronic supplementary material

Supplementary figure legends

Supplement, Figure 1

Supplement, Figure 2
