# Supplementary material for: Reduced expression of stearoyl-CoA desaturase-1, but not free fatty acid receptor 2 or 4 in subcutaneous adipose tissue of patients with newly diagnosed type 2 diabetes mellitus
Source: Nutr Diabetes. 2018 Sep 7;8:49. doi: 10.1038/s41387-018-0054-9 (PMC6127327; doi:10.1038/s41387-018-0054-9)
Supplement: Supplementary file 1 — Supplementary figure legends [file 41387_2018_54_MOESM1_ESM.pdf]

Supplement

Legends

Supplementary Figure 1. Metabolite plasma concentrations during mixed meal tolerance test (MMT) Plasma concentrations of plasma a) glucose, b) insulin, c) C-peptide, and d) non-esterified fatty acids (NEFA) during ingestion of a mixed meal in glucose-tolerant humans (CON; n=22-25) and in patients with type 2 diabetes mellitus (T2D; n=23-25) after mixed meal ingestion. Data are given as mean±SEM. Two-tailed Mann-Whitney U test was performed; *$p<0.05$, ** $p<0.01$, ***$p<0.001$.

Supplementary Figure 2. Western blot analysis of FFAR2, FFAR4 and SCD1 a) Protein expression levels of free fatty acid receptor 2 (FFAR2) (n=23 for Gel 1; n=22 for Gel 2 with 2 controls from Gel 1). b) Protein expression levels of free fatty acid receptor 4 (FFAR4) (n=45). c) Protein expression levels of stearoyl-CoA desaturase-1 (SCD1) (n=45). Kilodalton (kD).
