# Supplementary figures and images for: Reduced expression of stearoyl-CoA desaturase-1, but not free fatty acid receptor 2 or 4 in subcutaneous adipose tissue of patients with newly diagnosed type 2 diabetes mellitus
Source: Nutr Diabetes. 2018 Sep 7;8:49. doi: 10.1038/s41387-018-0054-9 (PMC6127327; doi:10.1038/s41387-018-0054-9)

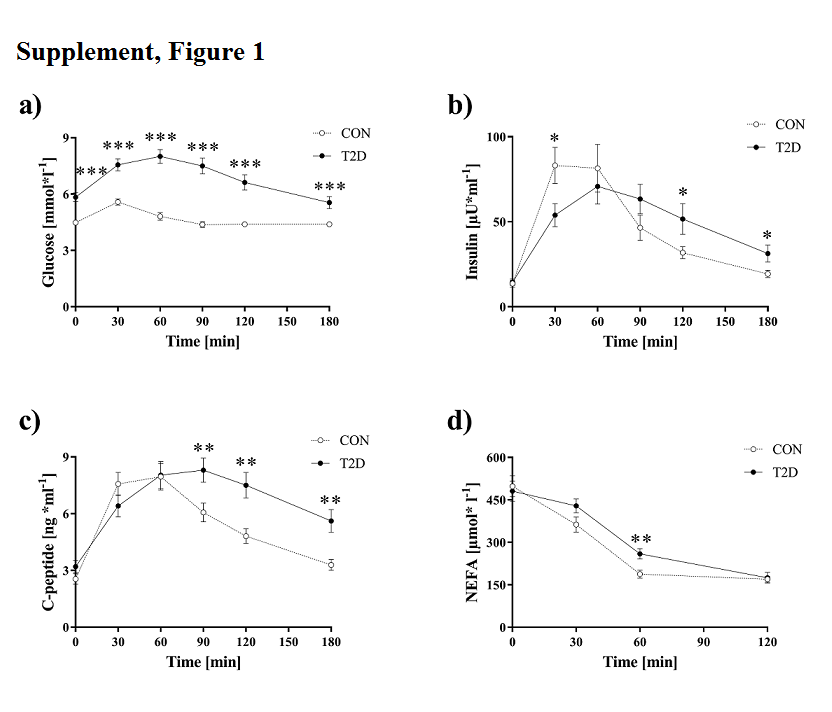

Supplement: Supplementary file 2 — Supplement, Figure 1 [file 41387_2018_54_MOESM2_ESM.tif]

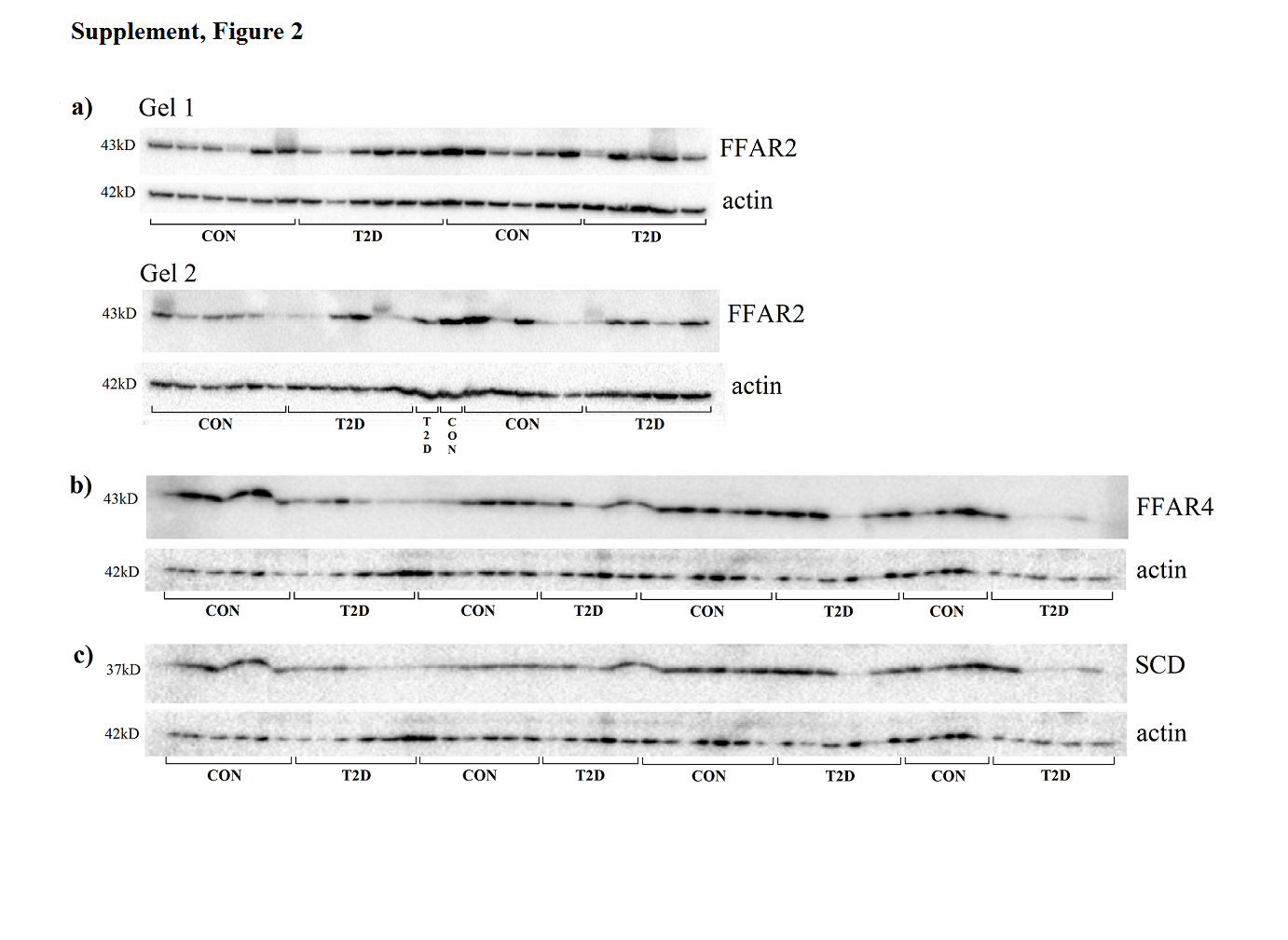

Supplement: Supplementary file 3 — Supplement, Figure 2 [file 41387_2018_54_MOESM3_ESM.tif]
